# Adolescents Caught Between the Temptation and the Habit of Smoking

**DOI:** 10.7759/cureus.83108

**Published:** 2025-04-28

**Authors:** Ana-Maria Dadulescu, Cristiana Susana Glavce, Suzana Turcu, Adriana Borosanu

**Affiliations:** 1 Medical Anthropology, Francisc I Rainer Institute of Anthropology, Bucharest, ROU

**Keywords:** adolescence, public health, risk factors, smoking, vulnerability

## Abstract

Background: Adolescence is a stage characterized by behaviors that pose significant risks to long-term health, and smoking is one of the most prominent risks among young people. This period is also considered a stage with an increased need for interaction and acceptance from peer groups, which can lead adolescents to take on new risks. In this context, smoking, in various forms (cigarettes, cigarillos, vape/e-cigarettes, chewing tobacco, etc.), is a major public health issue among young people. Tobacco use, perceived as a socializing factor, provides adolescents with the opportunity to integrate more easily into groups.

Objective: The objective of this research was to identify potential changes in adolescents' smoking behaviour during the COVID-19 pandemic, based on sociodemographic data. Additionally, we aimed to identify the existence of vulnerabilities that could lead to the later development of the smoking habit.

Material and methods: The research sample comprised 521 subjects aged 15-19 years, both girls and boys, from rural and urban areas in the southern (Olt) and northern (Suceava) regions of Romania. The study was cross-sectional and included data collected online through an anthropological questionnaire between April and May 2021. The study participants were enrolled in high school education conducted exclusively online during this period due to the context generated by the pandemic. The questionnaire included a series of questions related to smoking-related behaviours among adolescents, such as the age of onset of smoking temptation and the subsequent development of this habit, analyzed in relation to sociodemographic variables. Data were processed using IBM SPSS Statistics, version 26 (IBM Corp., Armonk, USA).

Results: The results were obtained from the analysis of data provided by the 521 subjects in the research sample. Approximately 45% of our study subjects were tempted and tried to smoke, but only 17.3% became regular smokers and smoked daily, while 4.2% continued to be occasional smokers (3-5 cigarettes/month). It was found that there were no statistically significant differences by gender, place of origin, or geographical area in the age at which adolescents were tempted and even tried smoking. About 52.5% of the adolescents who tried to smoke did not become regular smokers, while those who became regular or occasional smokers represented 47.5%. In our sample, the vulnerable age for the subsequent establishment of regular smoking behaviour was between 13 and 14 years. Adolescents who perceived their family income as high or average reported higher tobacco consumption compared to those who perceived their family income as low.

Conclusions: Tobacco consumption habits did not seem to be influenced by the new situation created by the COVID-19 pandemic. Regardless of gender, place of origin (rural or urban), or geographical region, the age at which the temptation to smoke was most likely to develop into a habit was 14 or younger. It is noteworthy that more than half of the adolescents were not tempted to experiment with smoking. Among those who tried tobacco and continued smoking, boys showed a higher percentage than girls. Furthermore, a correlation was observed between family income and the quantity of tobacco consumed.

## Introduction

Adolescence is perceived as a period during which the need for direct interaction and acceptance from the peer group becomes more pronounced [1]. Within the peer group, the openness to risky behaviours increases, among which smoking is one of the most common. Tobacco use is a facilitator of socialization and group integration and is often influenced by peers, friends, and family, especially if they smoke [2,3].

One of the most consumed substances during adolescence is tobacco [4]. The negative effects of tobacco use are manifold and can seriously affect quality of life [5]. Smoking exposes the body to more than 7,000 toxic substances, including 70 carcinogens that affect almost every organ system in the body [6]. Recent studies have identified a higher risk for smokers regarding COVID-19 infection and the severity of symptoms.

Adolescents start to smoke for various reasons (curiosity, spirit of adventure, integration into a group of friends) and tend to diminish the existence of risks and harmful effects on their health [7], which can lead to the adoption and maintenance of smoking behaviour. As with other categories of drugs, smoking causes neurobiological changes in the adolescent brain [8]. The age of onset of the temptation to smoke is becoming increasingly lower, thereby raising the risk that adolescents will later become smokers [9,10].

In 2017, data were collected from 12,328 subjects aged between 13 and 17 years from 11 European countries, with Romania being part of the study. Of the Romanian subjects analyzed, 21.5% were daily smokers, placing them below the European average for this category of 30.9% [11]. Regarding regular tobacco consumption, studies highlight that if a person has not become a smoker by the age of 25, they have a lower risk of becoming a smoker later in life [12,13]. 

The development of technology has transformed direct human-to-human interaction into a virtual reality, where unhealthy habits, including tobacco use, are often promoted (advertisements promoting tobacco and the new range of tobacco products, vaping devices, electronic cigarettes, heated cigarettes that do not contain tobacco but contain other harmful substances, etc.). This type of advertising targets vulnerable groups, including adolescents [14]. It has been noted that the socioeconomic status of the family, as well as the adolescent's perception of the family's financial condition, influences tobacco consumption [15]. Furthermore, early initiation of smoking has numerous repercussions on both physical and mental health, thus becoming a public health issue due to the increasing number of smokers globally [16].

In this context, the World Health Organization (WHO) [17], through the Global Youth Tobacco Survey (GYTS), provides data regarding tobacco use among adolescents aged 13-15 years. In Romania, the GYTS 2017 [18] reports that 14.6% of students (16.4% boys and 12.5% girls) consume some form of tobacco, marking an increase compared to the GYTS 2013 [19], which indicated that 11.2% of students (12.2% boys and 10.1% girls) were tobacco users.

In the Organization for Economic Cooperation and Development (OECD) report, Health at a Glance 2023 [20], it is mentioned that 18.9% of young people in Romania aged 15 and over are smokers, which is in line with the European average of young people who smoke. The 2019 report from the European Statistical Office (Eurostat) [21] includes data regarding Romanian youth aged 15-16 years, with 31% reporting that they had smoked in the past month, which is above the European average of 21% [22]. For the age group 15-19 years, the data shows that 6.9% smoke less than 20 cigarettes a day, while 1.4% smoke more than 20 cigarettes per day [21].

According to the 2019 report from the European School Survey Project on Alcohol and Other Drugs (ESPAD), the prevalence of smoking onset at the age of 13 or younger is 18% at the European level, while in Romania, it reaches 20%. Within the report, the average by gender is 20% for boys and 15% for girls. Adolescents in Romania have a higher rate of smoking initiation than the average of the 35 countries participating in the study, with 23% of boys and 17% of girls having started smoking for the first time at the age of 13 or earlier [23].

In a doctoral thesis presented in 2019, which focused on adolescents in Romania, including 10,114 subjects aged between 14 and 17 years, it was found that 44% were non-smokers, 52.9% reported themselves as smokers, 2% as e-cigarette smokers, and 1% did not respond. In relation to the entire study sample, the proportion of non-smokers was relatively equal by gender (boys: 42.5%, girls: 45%). The most frequent attempt to smoke was among 14-year-olds (17.1%), followed by 15-year-olds (13.1%). Regarding the number of cigarettes smoked daily, 18.9% reported smoking between 5-10 cigarettes, 16.7% smoked between 10-15 cigarettes, and 4.1% smoked more than 20 cigarettes [24].

In order to observe the extent to which adolescents' tobacco use behaviour was influenced by the context generated by the COVID-19 pandemic, it was necessary to provide a brief overview of national and international research conducted prior to this period. This approach will allow us to identify possible changes in adolescents’ smoking behaviour as a result of the social and educational reality imposed by the health crisis. 

## Materials and methods

Study design

The data used in this article were based on a quantitative study and were collected through an anthropological questionnaire administered online (Google Drive (Google LLC, Mountain View, USA)) during the COVID-19 pandemic period (April-May 2021). During this time, adolescents were required to stay with their family of origin, regardless of their place of origin (rural or urban), as all study participants were enrolled in high school education conducted exclusively online due to pandemic-related restrictions.

An excerpt of the questionnaire, including only items relevant to this study, is presented in the Appendices.

At the time the questionnaire was distributed, the participants were informed and gave their consent in accordance with legal regulations regarding personal data protection. Adolescents accessing the survey who stated they were under the age of 16 years were prompted to seek opt-out parental consent. They were directed to ask their parent/guardian to read some information about the study and were asked to tick a box to indicate they had done so before being permitted to proceed to the survey. The selection of study participants was voluntary, did not involve the risk of identifying individuals, and offered the possibility of withdrawal at any stage of completing the questionnaire. Respondents could complete the questionnaire at any time.

Participants and data collection

The participants were selected based on their voluntary agreement to participate in the study, geographical region, age range, and educational status (convenience sampling). A pilot test with 15 adolescents assessed the questionnaire's clarity and content, and the feedback collected improved the final version. 

Initially, 550 subjects responded, randomly selected among students belonging to a form of secondary education in the two counties (Olt and Suceava). The completion of the questionnaire was carried out at home, and only participants who completed the questionnaire in full were included in the sample. Thus, the study included 521 male (N=153) and female (N=368) adolescents aged 15-19 years, from rural (N=313) and urban (N=208) areas, from the Olt (South) (N=224) and Suceava (North) (N=297) regions of Romania. 

In this context, the study did not aim to evaluate the entire student population in the country; therefore, it is not representative, and the results obtained cannot be generalized.

Variables and measurements

The questionnaire, comprising 69 items, was used to collect sociodemographic data, information about the family, and to assess risk behaviours with health effects (tobacco use, alcohol consumption, energy drink consumption, diet, sedentary lifestyle, sleep duration). Time spent on social networks or using their mobile phones, leisure time activities, documented medical conditions, and body appearance self-perception were also investigated.

This study assessed modifications in adolescents' smoking behaviour during the COVID-19 pandemic and evaluated potential susceptibility to future smoking habits based on the responses regarding tobacco use received in the questionnaire (smoking initiation, smoking frequency).

Data analysis

To achieve the research objectives, inferential statistics (chi-squared test with a significance level of p < 0.05 and crosstabulation) were applied, and data processing was carried out using IBM SPSS Statistics, version 26 (IBM Corp., Armonk, USA) and Excel (Microsoft Corp., Redmond, USA).

Before conducting the research, approval was obtained from the Ethics Committee of the Francisc I Rainer Institute of Anthropology, Romanian Academy (254/2021). The participants were informed and gave their consent in accordance with legal regulations regarding personal data protection. The selection of study participants was voluntary, did not involve the risk of identifying individuals, and offered the possibility of withdrawal at any stage of completing the questionnaire.

## Results

**Figure 1:**
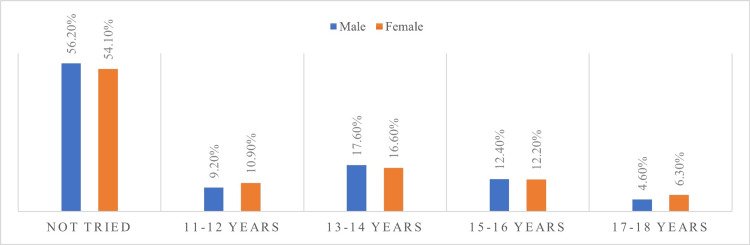
Age at which adolescents smoked their first cigarette, by gender

More than half of the adolescents aged 15-18 years old, both boys (M) and girls (F), stated that they were not tempted and did not attempt to smoke (M = 56.2%; F = 54.1%). Adolescents were tempted to experiment with smoking as early as 11-12 years old (M = 9.2%; F = 10.9%), but the majority first attempted smoking between the ages of 13-14 (M = 17.6%; F = 16.6%), followed by the 15-16 age group (M = 12.4%; F = 12.2%). A decrease in those attempting smoking for the first time was noted at the age of 17-18 (M = 4.6%; F = 6.3%) (Figure 1).

**Figure 2:**
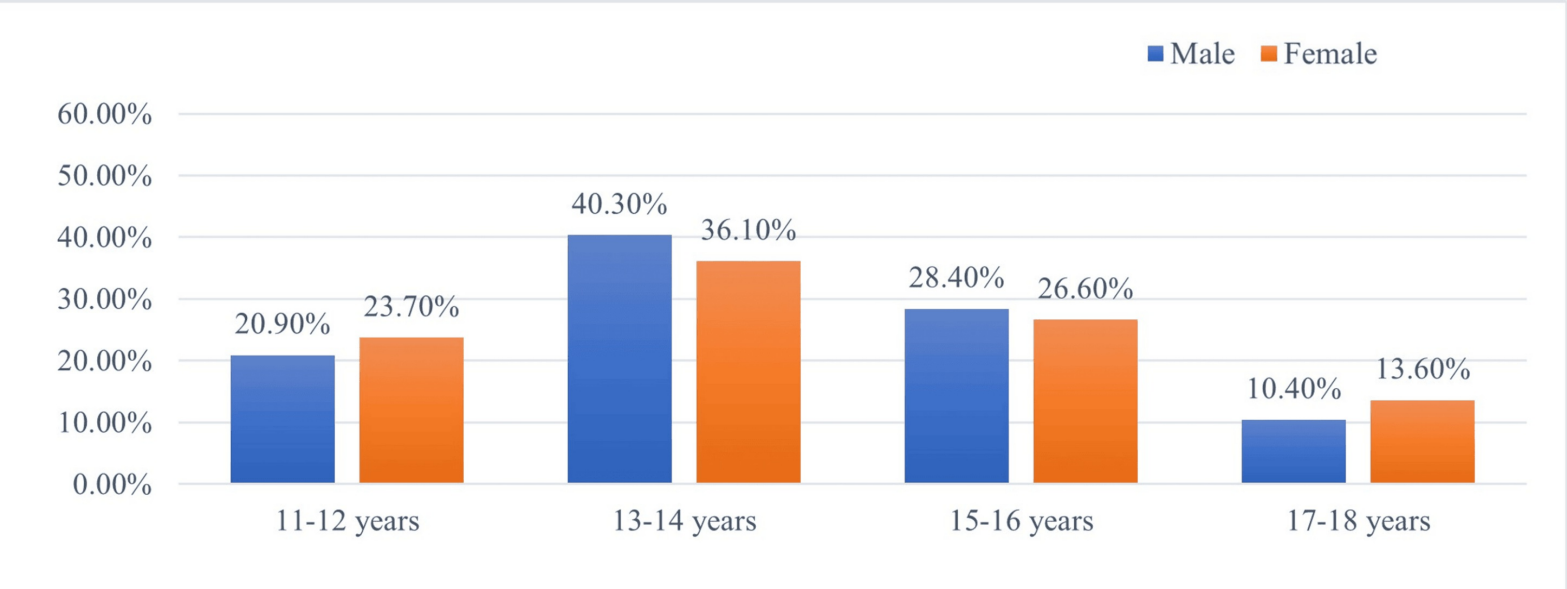
Age at which adolescents were tempted to smoke a cigarette, by gender

Among adolescents who had tried smoking, it was observed that the vulnerable age regarding the temptation to smoke was during the 13-14 age stage for both genders (M = 40.3%; F = 36.1%). The initiation of smoking behaviour in the studied sample began at the 11-12 age stage, which was more commonly encountered among girls (Figure 2).

**Figure 3:**
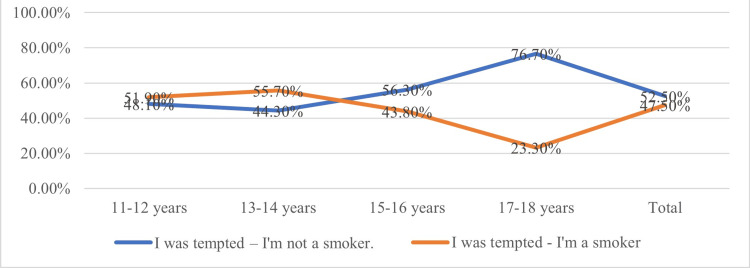
Temptation and consolidation of the smoking habit by age of first cigarette smoked

The results indicated a significant relationship between the age at which adolescents were tempted to smoke and their subsequent tendency to continue smoking. We found that adolescents who were tempted to smoke at younger ages (11-12 years and 13-14 years) were more likely to become smokers. The chi-squared statistical test confirmed that the younger the age of temptation to try smoking, the higher the risk of becoming a smoker (χ² = 2.0; p = 0.017, df = 3, N = 236). Thus, the results suggested a statistically significant correlation between the age at which adolescents were tempted to smoke and the stabilization of this behaviour. These criteria were marginally differentiated by the place of origin (rural/urban) and geographical area (Olt/Suceava) (Figure 3).

In the entire study sample, the subjects who had never tried smoking represented 54.7% (N = 285), while another proportion experimented with smoking without becoming regular smokers (23.8%, N = 124). The remaining subjects adopted smoking behaviour (21.5%, N = 112). Among the smoking subjects, some were only occasional smokers, consuming 3-5 cigarettes/month (19.6%, N = 22), others smoked up to four cigarettes/day (30.4%, N = 34), and half of them smoked more than five cigarettes/day (50%, N = 56). Regarding the subjects who tried smoking, some continued to be regular smokers (47.5%, N = 112), while others did not become smokers (52.5%, N = 124). From a gender dimorphism perspective, it was observed that boys tended to remain regular smokers more frequently than girls (M = 55.2%, F = 44.4%) (Table 1).

**Table 1:** Temptation to try and keep smoking, by gender

Temptation to smoke						
Gender	I was tempted, and I'm not a smoker		I was tempted, and I'm a smoker		Total	
	N	%	N	%	N	%
Male	30	44.8%	37	55.2%	67	100%
Female	94	55.6%	75	44.4%	169	100%
Total	124	52.5%	112	47.5%	236	100%

**Figure 4:**
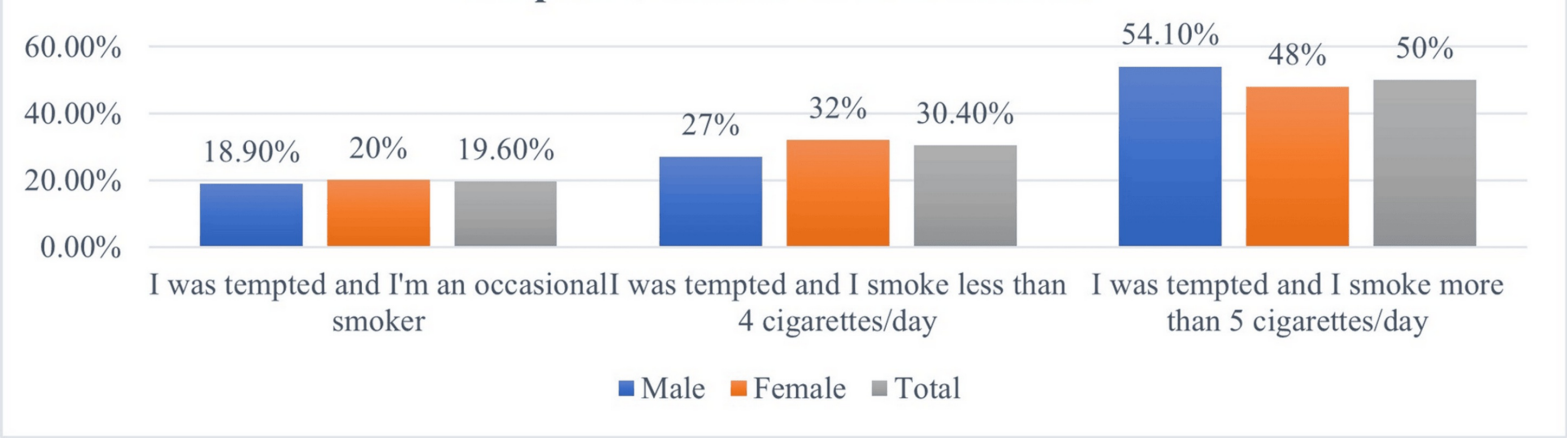
Amount of cigarettes consumed daily, by gender

Analyzing the quantity of cigarettes smoked, it was observed that 19.6% of individuals smoked occasionally (M = 18.9%; F = 20%), 30.4% smoked four cigarettes/day (M = 27%; F = 32%), and 50% smoked more than five cigarettes/day (M = 54.1%; F = 48%). Thus, boys were more frequent smokers of more than five cigarettes/day, while girls were more likely to be occasional smokers or smoked up to five cigarettes/day (Figure 4).

**Figure 5:**
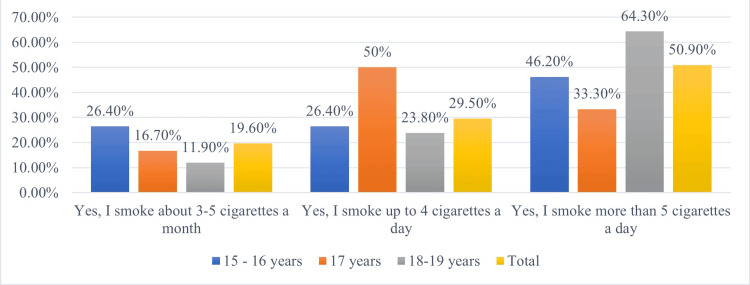
Proportion of smokers, by age

In analyzing adolescents who smoked more than five cigarettes a day, a consistent upward trend was observed as age increased. At 15-16 years, 46.2% represented all smokers, while at 18-19 years, this proportion rose to 64.3%. An exception to this trend was seen among 17-year-olds (50%), who limited their consumption to four cigarettes a day (Figure 5).

**Figure 6:**
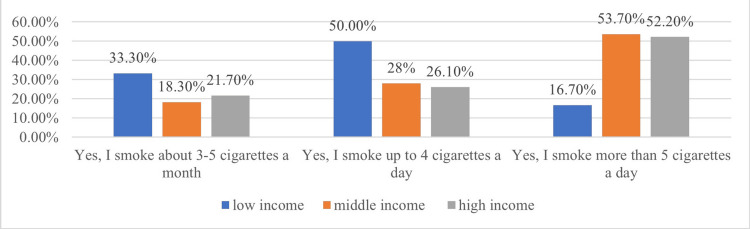
Smoking habit and family income

More than half of adolescents who perceived themselves as coming from families with average (A) and high (H) incomes smoked more than five cigarettes/day (A = 53.7% and H = 52.2%), while those who considered their family income to be low (L) were predominantly regular smokers of up to four cigarettes/day (L = 50%) and occasional smokers consuming 3-5 cigarettes/month (L = 33.3%) (Figure 6).

## Discussion

An important aspect is that nicotine dependence develops more quickly and after less cigarette consumption in young people than in adults [25]. This puts adolescents who experiment with smoking, even occasionally, at considerably higher risk of becoming daily smokers.

An early onset of smoking significantly increases adolescents' risk of developing addiction and continuing to smoke in the long term. According to the ESPAD report, the age of smoking onset is 13 years or earlier for 18% of young people in Europe, while in Romania, this figure reaches 20%. Adolescents in Romania have a higher rate of smoking initiation compared to the average of the 35 countries that participated in the study, with 23% of boys and 17% of girls smoking for the first time at the age of 13 or earlier [23]. Several recent studies indicate that a substantial proportion of young people smoke their first cigarette before the age of 14: the Global Youth Survey shows that 32% of school children smoked their first cigarette before the age of 14 [26], and successive studies conducted in Romania report that 56% of young smokers first smoked around the age of 12 [27]. In our study, we found that the proportion of adolescents experimenting with tobacco use at the age of 14 or earlier was 26.8% for boys and 27.5% for girls, compared to those who tried smoking at the age of 15 or later, with 17% of boys and 18.4% of girls, respectively.

Comparing the age stages, we found that subjects started smoking at 11-12 years (10.4%), 13-14 years (16.9%), 15-16 years (12.3%), and 17-18 years (5.8%). The results are relatively similar to those obtained in the doctoral thesis [24] defended at the doctoral school of the Romanian Academy in 2019, where at the age of 12, 7.1% tried smoking, at 13 years 11.7%, at 14 years 17.1%, at 15 years 13.1%, at 16 years 8.6%, and at ages over 16 years 11%. In our research group, smokers of various categories represented 21.4% in 2021, which is similar to the results of a study conducted in 11 European countries, including Romania, in 2017, i.e., 21.5% [11]. We were also close in terms of the results of the OECD studies conducted in 2019 (18.9%) [20].

We were interested in whether adolescents have the desire (temptation) to try smoking and whether all those who try smoking become regular smokers. We observed that 45.3% were tempted to experiment with smoking, but only 21.5% declared themselves as regular smokers, while 23.8% had only tried. The subjects who had not experimented with smoking at all represented 54.7%.

The GYTS study provides relevant data regarding tobacco use among adolescents in Romania, indicating that 14.6% of students consume tobacco (M = 16.4%, F = 12.5%) [19]. Another study highlights tobacco consumption among adolescents aged 15-19, with 8.3% of them smoking daily, 6.9% smoking fewer than 20 cigarettes/day, and 1.4% smoking more than 20 cigarettes/day [21]. With regard to the quantity of tobacco consumed, the reported figures refer exclusively to the behaviour of adolescent smokers, in contrast to previous studies, which considered the entire adolescent sample, including both smokers and non-smokers. The results showed that 19.6% smoked occasionally (M = 18.9%, F = 20%), 30.4% smoked up to four cigarettes per day (M = 27%, F = 32%), and 50% consumed more than five cigarettes per day (M = 54.1%, F = 48%). These data suggest that boys are more likely to consume larger quantities of tobacco daily, while girls are more inclined to smoke occasionally or in smaller quantities.

We observed changes in smoking behaviour by age that revealed a steady upward trend in the percentage of those who smoked more than five cigarettes/day as age increases. Thus, at the age of 15-16, they accounted for 46.2% of all smokers, while at the age of 18-19, this rose to 64.3%. An exception to this trend was among 17-year-olds, 50% of whom limited their consumption to four cigarettes/day.

Studies clearly indicate that a younger age at the time of the first cigarette is a significant risk factor for developing nicotine addiction and continuing to smoke throughout adolescence and into adulthood. The results obtained in our research suggest that the age at which young people experience the temptation to smoke is significantly associated with their subsequent smoking behavior, with the most vulnerable age being 13-14 years, which marks the onset of adolescence. This highlights the importance of smoking prevention through early interventions (such as anti-tobacco campaigns and health education), which should address adolescents' motivation not to experiment with smoking.

The restrictions generated by the COVID-19 pandemic seem to have reduced tobacco consumption in our study compared to the data from the previously mentioned doctoral thesis. This reduction could be due to the fact that young people spent more time with their families and the influence of the peer group diminished [1]. Compared to the official Eurostat or OECD data, the percentage of teenagers smoking during the mentioned period (which also includes a crisis situation) seems to be similar, or perhaps slightly higher, in the results of our study.

Study limitations

The research sample focused on a group of high school students characterized by their commitment to study and financial support from their families. In this context, the study did not aim to evaluate the entire adolescent population in the counties of Olt and Suceava; therefore, it is not representative, and the results obtained cannot be generalized. Additionally, causality cannot be determined based on this study. The study was conducted during the COVID-19 pandemic, with no subsequent follow-up to assess whether the results hold in a normal context. The data were self-reported through an online questionnaire, which may introduce potential errors in the participants' assessment of the information and may be subject to self-reporting bias.

Recommendations for further research

Further studies are needed to replicate the research in order to validate the results, including other categories of adolescents in the context of their usual lives, unrestricted by crisis-related circumstances.

## Conclusions

The study highlights that more than half of adolescents, regardless of gender, are not tempted to experiment with smoking. Boys continue to smoke at a higher rate than girls, and adolescents from families with medium and high incomes tend to consume greater amounts of tobacco compared to those from low-income families. Additionally, the results indicate that most adolescents who try smoking at the age of 14 or younger are more likely to become long-term smokers. Regarding the impact of the restrictions imposed by the COVID-19 pandemic on tobacco use, the study did not identify significant differences in adolescent smoking behavior compared to studies conducted prior to the health crisis at the European level. However, a decrease in tobacco consumption was observed in comparison with data from a study conducted on Romanian adolescents. These results suggest that the limitation of social interactions did not significantly influence smoking behavior during that period.

## Appendices

Gender: a. Female      b. Male Age:........................... County:....................... Place of origin: a. Rural          b. Urban

Do you consider yourselves a family with income? a. Low income      b. Middle income        c. High income

Here are a few questions that aim to explore your preferences related to consumption and habits.

The first cigarette was smoked at the age of? a. 10 ani         b. 11            c. 12            d. 13              e. 14             f. 15          g. 16            h. 17          i. Over 17 years       j. I haven't tried

Do you usually smoke? (If you smoke, select the answer that is appropriate for you): a. No b. Yes, I smoke about 3-5 cigarettes a month c. Yes, I smoke less than 4 cigarettes a day d. Yes, I smoke around 5-10 cigarettes a day e. Yes, I smoke 10-15 cigarettes a day

What type of cigarette do you smoke? a. Regular cigarette            b. Electronic cigarette          c. IQOS             d. Hand-rolled cigarette

Do you smoke tobacco mixed with other substances? a. No b. I tried it once for sure c. Yes, I smoke 1-2 times a month d. Yes, I smoke 2-3 times a week e. Yes, I smoke every day

What is your opinion about illegal drugs? (marijuana, cocaine, heroin, opium, synthetic drugs) a. They are dangerous         b. I have tried them        c. I'd like to give up       d. They are not dangerous        e. Would like to try     

## References

[1] Andrews JL, Foulkes L, Blakemore SJ (2020). Peer influence in adolescence: public-health implications for COVID-19. Trends Cogn Sci.

[2] Park HK (1967). The social-politic consideration about youngmen’s drinking and smoking. J Korean Med Assoc.

[3] Park SH (2011). Smoking and adolescent health. Korean J Pediatr.

[4] Schmitz N, Kruse J, Kugler J (2003). Disabilities, quality of life, and mental disorders associated with smoking and nicotine dependence. Am J Psychiatry.

[5] da Silva Brito AL, Hardman CM, de Barros MV (2015). Prevalence and factors associated with the co-occurrence of health risk behaviors in adolescents. Rev Paul Pediatr.

[6] (2025). Health effects. https://tobaccoatlas.org/challenges/health-effects.

[7] Halpern-Felsher BL, Biehl M, Kropp RY, Rubinstein ML (2004). Perceived risks and benefits of smoking: differences among adolescents with different smoking experiences and intentions. Prev Med.

[8] Salmanzadeh H, Ahmadi-Soleimani SM, Pachenari N, Azadi M, Halliwell RF, Rubino T, Azizi H (2020). Adolescent drug exposure: a review of evidence for the development of persistent changes in brain function. Brain Res Bull.

[9] (2016). E-cigarette use among youth and young adults: a report of the surgeon general. https://www.cdc.gov/tobacco/data_statistics/sgr/e-cigarettes/pdfs/2016_sgr_entire_report_508.pdf.

[10] Wang TW, Gentzke AS, Creamer MR (2019). Tobacco product use and associated factors among middle and high school students - United States, 2019. MMWR Surveill Summ.

[11] Banzer R, Haring C, Buchheim A (2017). Factors associated with different smoking status in European adolescents: results of the SEYLE study. Eur Child Adolesc Psychiatry.

[12] Flor LS, Reitsma MB, Gupta V, Ng M, Gakidou E (2021). The effects of tobacco control policies on global smoking prevalence. Nat Med.

[13] Li X, Borodovsky JT, Kasson E, Kaiser N, Riordan R, Fentem A, Cavazos-Rehg PA (2021). Exploring how tobacco advertisements are associated with tobacco use susceptibility in tobacco naive adolescents from the PATH study. Prev Med.

[14] Diaz MC, Kierstead EC, Edwards D (2022). Online tobacco advertising and current chew, dip, snuff and snus use among youth and young adults, 2018-2019. Int J Environ Res Public Health.

[15] Hammond MA, Khurana A, Stormshak EA (2021). Adolescent measures of family socioeconomic status: reliability, validity, and effects on substance use behaviors in adolescence and young adulthood. Prev Med Rep.

[16] (2025). OECD indicators. https://www.oecd.org/en/data/indicators.html?orderBy=mostRelevant&page=0.

[17] (2025). WHO Report on the Global Tobacco Epidemic 2019: Offer Help to Quit Tobacco Use. WHO Report on the Global Tobacco Epidemic, 2019.

[18] (2024). 2017 GYTS fact sheet Romania. https://www.who.int/publications/m/item/2017-gyts-fact-sheet-romania.

[19] (2025). Global youth tobacco survey. https://www.who.int/teams/noncommunicable-diseases/surveillance/systems-tools/global-youth-tobacco-survey?utm_medium=email&utm_source=transaction.

[20] (2023). Health at a Glance 2023: OECD Indicators. Health at a Glance.

[21] (2024). Daily smokers of cigarettes by sex, age and educational attainment level. https://doi.org/10.2908/HLTH_EHIS_SK3E.

[22] (2023). Romania: country health profile 2023, state of health in the EU. OECD/European Observatory on Health Systems and Policies.

[23] ESPAD Group (2025). ESPAD Report 2019: Results From the European School Survey Project on Alcohol and Other Drugs. https://www.espad.org/espad-report-2019.

[24] Mihai C (2019). Health Status and Consumption of Psychoactive Substances Among Young People in Romania. https://rei.gov.ro/index.php?&ddpN=917523103&we=7de50869e17bec77664920c1aeae1a47&wf=dGFCall&wtok=&wtkps=jVHNcoMgEH6VDufGCIjoeslMzu1kJn0BCkSJij9o2kwm714wTg495bbL97e7CEjh5oAB+pnqxqHis3BAATmjkK8YATR2zfg7Vsrm54kMNBN5K/qrvVwGOpVz01/PfWk2uGV1pk4bcjK2ZEHrpbsJFcLb3ZZOkdCR0NEASiErvf06kl2OOSMUxxQa4yZwWoyyCh6UA+pdFEcvkQHBbEKF00WW4DzhaZryaHm/ewDQMhJ+fYiV/FAawDxhDGPG4+L+MGyfHO9XD4GUJfkK+/mV6u3huMUZ5QlmGc2X48SAnoHv/zD/GXpZyG/UdmpudNQKYyNpxaTkHKnOiVEf5u/GSL0qQrDPSwDtu15ILez89mEq4df+Aw==&wchk=d6fa2ce4b897ad82c400a93ec9f41e72e887f342.

[25] Bucuresti CP (2015). Smoking among adolescents: intervention program (Article in Romanian). https://ultrapsihologie.ro/2015/02/16/fumatul-in-randul-adolescentilor-program-de-interventie/.

[26] (2025). Smoking and tobacco use. https://www.cdc.gov/tobacco/global/index.htm.

[27] Mihalache F, Neguţ A, Tufă L (2020). Child Welfare in Rural Areas 2020. Child Welfare in Rural Areas 2020.

